# Is Non-Contrast CT Adequate for the Evaluation of Hepatic Metastasis in Patients Who Cannot Receive Iodinated Contrast Media?

**DOI:** 10.1371/journal.pone.0134133

**Published:** 2015-07-28

**Authors:** Han Bum Jee, Min Jung Park, Hye Sun Lee, Mi-Suk Park, Myeong-Jin Kim, Yong Eun Chung

**Affiliations:** 1 Department of Radiology, Severance Hospital, Research Institute of Radiological Science, Yonsei University College of Medicine, Seoul, Republic of Korea; 2 Department of Radiology, Ajou university school of medicine, Suwon, Republic of Korea; 3 Biostatistics Collaboration Unit, Department of Research Affairs, Yonsei University College of Medicine, Seoul, Republic of Korea; University of Munich, GERMANY

## Abstract

**Objective:**

To evaluate the appropriateness of follow-up with only non-enhanced CT (NECT) in patients with gastrointestinal cancer.

**Subjects and Methods:**

This retrospective study included 323 patients with colorectal and gastric cancer who underwent two consecutive CT examinations (CT1 and CT2), including non-contrast and portal venous phase CT images, with an interval of 1 year. Patients were divided into 2 groups: Group A included patients with no hepatic metastasis on CT1 and with or without newly developed metastasis on CT2 to evaluate the diagnostic performance of NECT for detecting newly developed hepatic metastasis; Group B included patients with known hepatic metastasis both on CT1 and CT2 to evaluate the accuracy of NECT for the assessment of hepatic metastasis based on RECIST criteria (version 1.1). Contrast-enhanced CT (CECT) images were considered as reference standards.

**Results:**

Group A included 172 patients (M:F = 107:65; mean age, 62.6 years). Among them, 57 patients had 95 metastases (mean size, 2.2 ± 1.3 cm). Per patient and per lesion sensitivity for diagnosing newly developed hepatic metastasis was 56.1–66.7% and 52.6–56.8%, respectively. In terms of small metastases (<1.5 cm), per lesion sensitivity was significantly decreased to 28.1–34.4% (P < 0.05). Metastasis size measurements were significantly smaller on NECT (P < 0.001) compared with reference standards. In Group B, the accuracy of response evaluation based on RECIST criteria was 65.6–72.2%.

**Conclusions:**

NECT showed inadequate diagnostic performances in both detecting newly developed hepatic metastasis and evaluating the response of hepatic metastasis based on RECIST criteria.

## Introduction

The liver is one of the most common sites of metastasis from extrahepatic neoplasms because of high blood flow from the dual arterial and portal venous systems, and because it has a microscopic structure that is conducive to the lodgment of circulating tumor cells and a microscopic environment that favors the rapid growth of established tumor cells [1]. Early detection of hepatic metastasis or accurate evaluation of preexisting hepatic metastasis is essential to make treatment plans and to predict prognosis [2]. Contrast-enhanced computed tomography (CECT) is widely used as the primary imaging modality for the evaluation of hepatic metastasis, and overall sensitivity has been reported as approximately 72–83% with recent multi-detector CT systems [3,4].

Because most hepatic metastatic lesions, including those from colorectal and gastric cancer, are hypovascular compared with adjacent normal liver parenchyma, they are usually evaluated in the portal venous phase (PVP) in which the liver parenchyma shows peak enhancement, resulting in maximization of tumor-to-liver contrast differences [5,6]. However, NECT is frequently performed without administration of contrast media (CM) in patients with impaired renal function, who are vulnerable to contrast-induced nephropathy (CIN). Because development of CIN increases in-hospital mortality up to 20% and is also a predisposing factor in the long-term loss of kidney function [7], hesitation about the use of iodinated CM in patients with impaired renal function is understandable. Alternative imaging modalities can be used, such as non-contrast magnetic resonance imaging (MRI) and ultrasound (US), and ^18^F-FDG PET in these patients. However, limited accessibility, expense, longer scanning times of MRI and ^18^F-FDG PET, and lower sensitivity and operator dependency of US limit the general use or entire substitution of non-contrast MRI or US in the diagnosis of hepatic metastasis. Recently, contrast-enhanced US (CEUS) proved to be useful in the evaluation of hepatic metastases without nephrotoxicity [8]. However, CEUS is also operator dependent, needs dedicated equipment and has additional cost. Furthermore, CEUS has not been approved yet for the diagnosis of hepatic metastasis in some countries.

A prior study [9] reported that the diagnostic results of NECT and CECT regarding the status of liver metastasis concurred in 71% of cases. However, this study was performed before the era of multi-detector CT or the establishment of modern injection protocols for CM. According to the recent American College of Radiology (ACR) Appropriateness Criteria, NECT is usually not appropriate as a surveillance tool following treatment of the primary tumor but can be potentially indicated if patients cannot receive CM [6]. RECIST 1.1 criteria state that the appropriateness of NECT in the evaluation of patients who cannot receive CM should be carefully discussed with a radiologist on the basis of the tumor type and anatomic location of the disease, either prior to enrollment or after obtaining baseline CECT. Considering the rapidly increasing prevalence of chronic renal disease in the present aging society, the diagnostic performance of NECT for the evaluation of hepatic metastasis should be reevaluated as more and more patients undergo MDCT. Hence, the purpose of this study was to evaluate the appropriateness of follow-up with only NECT in patients with gastrointestinal cancer.

## Materials and Methods

The institutional review board of our institution (Yonsei University College of Medicine) approved this retrospective study and informed consent was waived. Patients’ information was anonymized and de-identified prior to analysis.

### Study Population

The inclusion criteria were as follows: 1) patients, including those who had and had not undergone surgery, with histologically proven colorectal cancer or gastric cancer; 2) patients with abdominal CECT images including non-contrast images and portal venous phase images (CT2); 3) patients who had undergone regular follow-up at our hospital for at least 1 year before and after CT2 and had prior CT (CT1) that had been performed within the previous 12 months. Patients who had undergone local treatment of the liver metastasis between the two CT scans, such as radiofrequency ablation (RFA) or trans-arterial chemoembolization (TACE), were excluded. Patients were divided into 2 groups: Group A and Group B. Group A included both patients with newly developed hepatic metastasis on CT2 that did not present on CT1 and patients without hepatic metastasis at both CT1 and CT2 for the evaluation of the diagnostic performance of NECT for detecting newly developed hepatic metastasis; Group B included patients with known hepatic metastasis on CT1 for the evaluation of the diagnostic performance of NECT according to the RECIST 1.1 criteria [10]. Among patients who belonged to Group B, patients who did not have target lesions in the liver (i.e., all hepatic metastatic lesions were <10 mm) were additionally excluded.

### Imaging Technique

CT scans were performed with multi-detector CT scanners (Somatom Sensation 64 or Definition Flash; Siemens Medical Solutions, Forchhein, Germany). A non-contrast image was obtained before administration of CM. Then, CM was injected by power injector via the antecubital vein at the rate of 2 mL/kg over 30 seconds. Using a bolus tracking technique, PVP was obtained 55 seconds after the Hounsfield unit (HU) value of the abdominal aorta had increased by 100 HU compared with baseline or 30 seconds after the end of the late arterial phase (LAP). CT parameters were as follows: 0.5-second rotation time; 120 kV; reference mAs, 240 mAs with automated tube current modulation; beam collimation, 0.6 mm; beam pitch, 1; and 3 mm slice thickness.

### Imaging Analysis

Two experienced abdominal radiologists (M.J.P with 4 years’ and Y.E.C with 7 years’ experience in abdominal imaging) independently reviewed NECT images of Group A (session 1) and B (session 2) in separate sessions. The reviewers knew that patients in Group A did not have metastasis on CT1 regardless of there being newly developed metastasis on CT2 and that patients in Group B already had liver metastasis on CT1, but they were not aware of any other clinical information. In the first session, the reviewers were asked whether there was newly developed metastasis on CT2. If there was newly developed metastasis, the reviewers were asked to determine the locations of each metastatic lesion and the largest diameter of each lesion with measurements going up to five lesions in order of increasing size. Then, the reviewers were asked to grade the confidence level of the lesion on a five-point scale: 1, very unlikely metastasis; 2, unlikely metastasis; 3, likely metastasis; 4, very likely metastasis; and 5, definitely metastasis [11]. Lesions with confidence levels of 3, 4, or 5 were regarded as metastases. In Group B, the reviewers were asked to measure the two largest liver metastases (as target lesions) in both the prior and the present CT images and determine response to chemotherapy based on the RECIST 1.1 criteria [10] as follows: Complete Response (CR), disappearance of all target lesions; Partial Response (PR), at least a 30% decrease in the sum of the diameters of the target lesions; Progressive Disease (PD), at least a 20% increase in the sum of the diameters of the target lesions or the appearance of one or more new lesions; Stable Disease (SD), neither sufficient shrinkage to qualify for PR nor sufficient increase to qualify for PD.

### Reference Standard

In Group A, all imaging modality findings, laboratory results, and clinical diagnoses were carefully reviewed at least 1 year before and after CT2 to determine the presence or absence of hepatic metastasis at CT2. In Group B, the largest two lesions were measured in HVP images both on CT1 and CT2, and the sum of the two measurements was calculated. Then, response was evaluated on the basis of RECIST 1.1 criteria [10]. All analyses of reference standards were performed by two experienced radiologists (M.J.K with 25 years’ and M.S.P with 15 years’ experience in abdominal radiology) who did not participate in the image analysis of the NECT images.

### Statistical Analysis

Diagnostic performances were assessed by frequency table and GEE approach. Tumor size according to the reference standard and reviewers’ measurement was compared by paired t-test. Interobserver agreement was evaluated by means of Cohen’s κ statistic. Degree of agreement was considered almost perfect, substantial, moderate, fair, and slight according to κ values between 0.81 and 1.00, between 0.61 and 0.80, between 0.41 and 0.60, between 0.21 and 0.40, and between 0.00 and 0.20.[12] All statistical analysis was performed by a biostatistician (H.S.L) using SAS (version 9.2; SAS Inc., Cary, NC, USA). P < 0.05 was considered statistically significant.

## Results

### Patients

**Fig 1:**
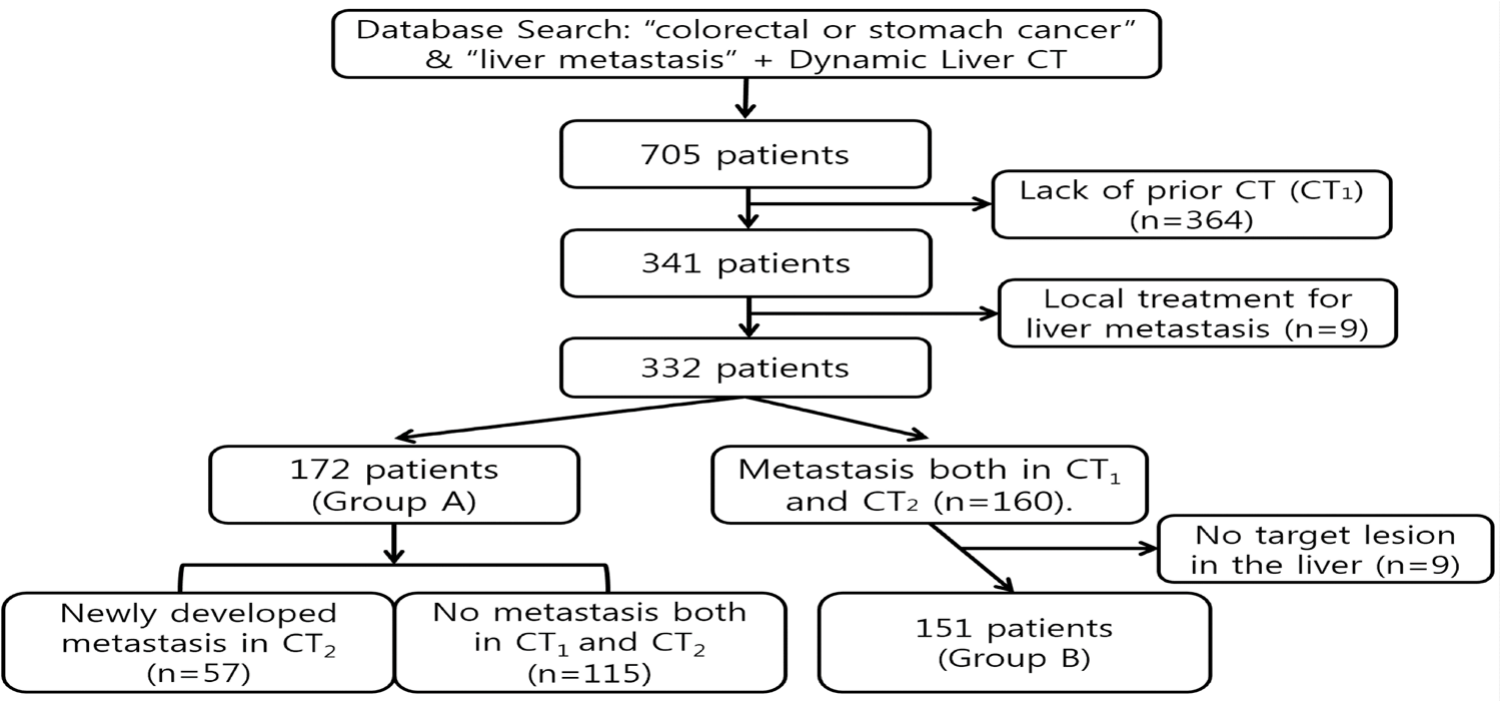
Flow chart of patients’ inclusion and subgrouping.

Through the careful review of medical records, 705 patients were identified from February 2005 through February 2012. Among them, 364 patients were excluded after review of CT images because no CT1 images were available within the preceding year, and 9 patients were excluded because TACE or RFA was performed between CT1 and CT2. Among the remaining 331 patients, 172 patients (107 men and 65 women; mean age, 62.6 years; age range, 33–85 years) were classified as Group A. In Group A, 57 patients had newly developed metastasis at CT2. Because an additional 9 patients were excluded because of the absence of target lesions in the liver, 151 of 160 patients were finally included in Group B (105 men and 46 women; mean age, 63.4 years; age range, 35–82 years) (Fig 1).

### Imaging Analysis

**Fig 2:**
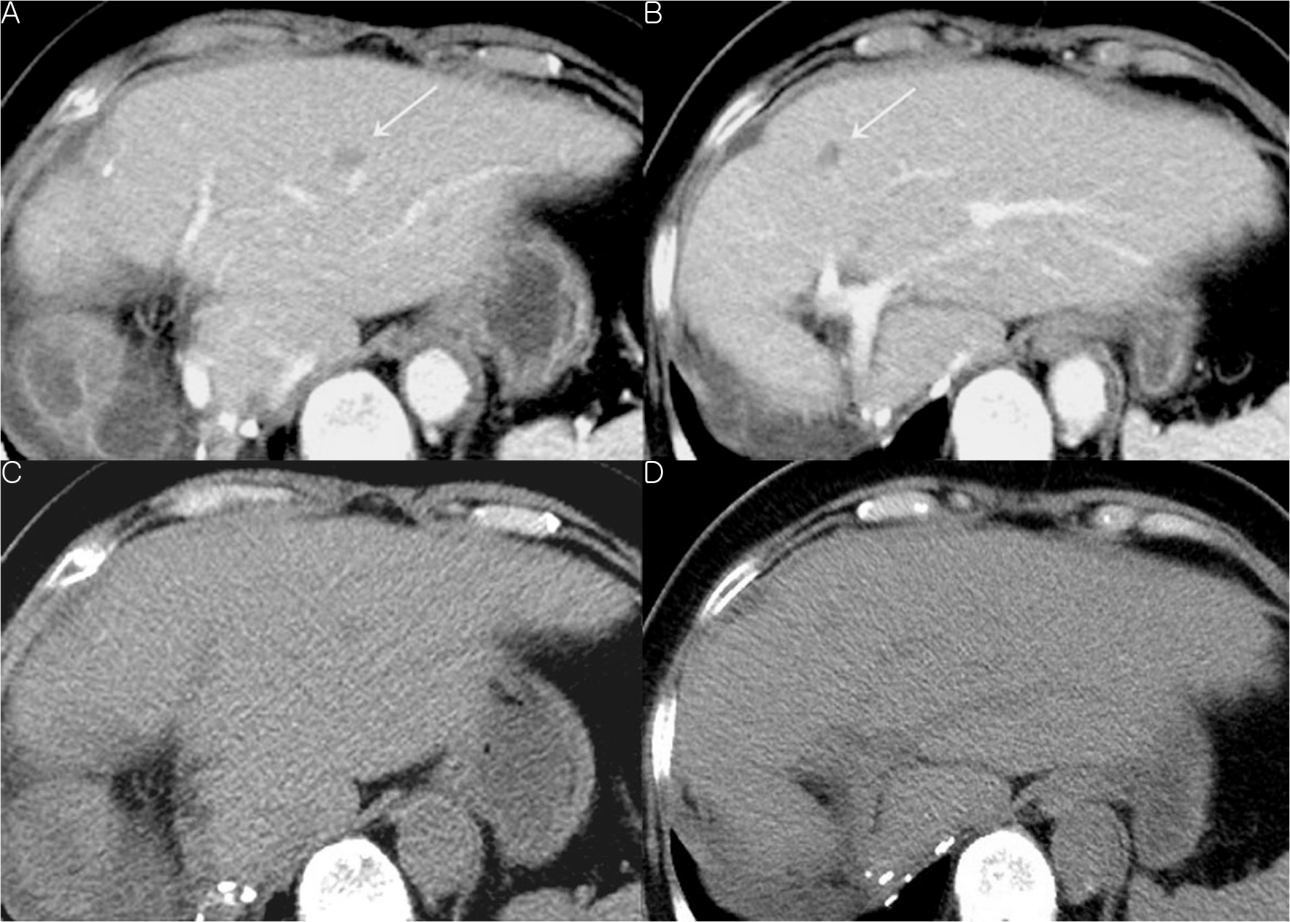
Seventy-year-old woman who underwent right hemicolectomy and right hemihepatectomy because transverse colon cancer with liver metastasis. (A, B) On follow-up CT, two newly developed metastases are seen in the remaining left lobe of the liver (arrows). (c, d) On non-enhanced CT images, low attenuating lesions were suspected at the same locations as those on contrast-enhanced CT, but neither of the reviewers could detect these lesions.

In Group A, there were 95 metastases (mean size = 21.7 ± 13.3 mm) in 57 patients. In per patient analysis of Group A, sensitivity and specificity was 56.1% and 92.2% for reviewer 1 and 66.7% and 98.3% for reviewer 2, respectively (Table 1). There was no statistically significant difference in sensitivity and specificity between the two reviewers (P > 0.05). Interobserver agreement between reviewers was substantial (κ = 0.661, 95% CI: 0.528–0.794). In terms of per lesion analysis, sensitivity was 52.6% for reviewer 1 and 56.8% for reviewer 2. Interobserver agreement was also substantial (κ = 0.606, 95% CI: 0.510–0.702) (Table 2). Among 95 newly developed metastases, 32 nodules were less than 15 mm in size. Diagnostic performance for smaller lesions less than 15 mm was significantly lower than that for all lesions with both reviewers (Table 2) (Fig 2). In terms of size measurement, size of hepatic metastatic lesions measured by each of the reviewers was significantly smaller than that of the reference standard both for all lesions and for lesions less than 15 mm (Table 3).

**Table 1:** Per patient analysis of diagnostic performance in detection of newly developed hepatic metastasis (Group A).

Per patient	SEN (95% CI)	SPE (95% CI)	ACC (95% CI)	PPV (95% CI)	NPV (95% CI)
**Reviewer 1**	56.1 (43.3–69.0)	92.2 (87.3–97.1)	80.2 (74.1–86.2)	78 (65.4–90.7)	80.9 (74.2–87.6)
**Reviewer 2**	66.7 (54.4–78.9)	98.3 (95.9–100.7)	87.8 (82.9–92.7)	95 (88.2–101.8)	85.6 (79.6–91.6)
**P value**	0.052	0.052	0.004	0.010	0.011

Note: Numbers in parentheses are 95% confidence intervals.

**Table 2:** Per lesion analysis of diagnostic performance in detection of newly developed hepatic metastasis (Group A).

		SEN (95% CI)	SPE (95% CI)	ACC (95% CI)	PPV (95% CI)	NPV (95% CI)
**Reviewer 1**	**All lesions**	52.6 (42.6–62.7)	97.5 (96.6–98.4)	93.9 (92.6–95.3)	64.1 (53.5–74.7)	96 (94.9–97.1)
	**Lesions <15 mm (n = 32)**	34.4 (17.9–50.8)	97.5 (96.6–98.4)	95.7 (94.5–96.9)	28.2 (14.1–42.3)	98.1 (97.3–98.9)
	**P value**	0.015	>0.999	<0.001	<0.001	<0.001
**Reviewer 2**	**All lesions**	56.8 (46.9–66.8)	98.7 (98.1–99.4)	95.4 (94.3–96.6)	79.4 (69.8–89.0)	96.4 (95.3–97.5)
	**Lesions <15 mm**	28.1 (12.5–43.7)	98.7 (98.1–99.4)	96.8 (95.7–97.8)	39.1 (19.2–59.1)	97.9 (97.1–98.8)
	**P value**	<0.001	>0.999	<0.001	<0.001	<0.001

Note: Numbers in parentheses are 95% confidence intervals.

**Table 3:** Comparison of size of the metastatic nodules in NECT and the reference standard.

		Size (mean ± SD)	P value (between reference standard and reviewers)	P value (between reviewers)
**All lesions (n = 95)**	**Reference standard**	21.7 ± 13.3		
	**Reviewer 1**	12.8 ± 15.8	<0.001	
	**Reviewer 2**	16.1 ± 18.1	<0.001	0.374
**Lesions <15 mm (n = 32)**	**Reference standard**	10.4 ± 2.3		
	**Reviewer 1**	6 ± 9.6	<0.001	
	**Reviewer 2**	4.3 ± 7.3	<0.001	0.423

**Fig 3:**
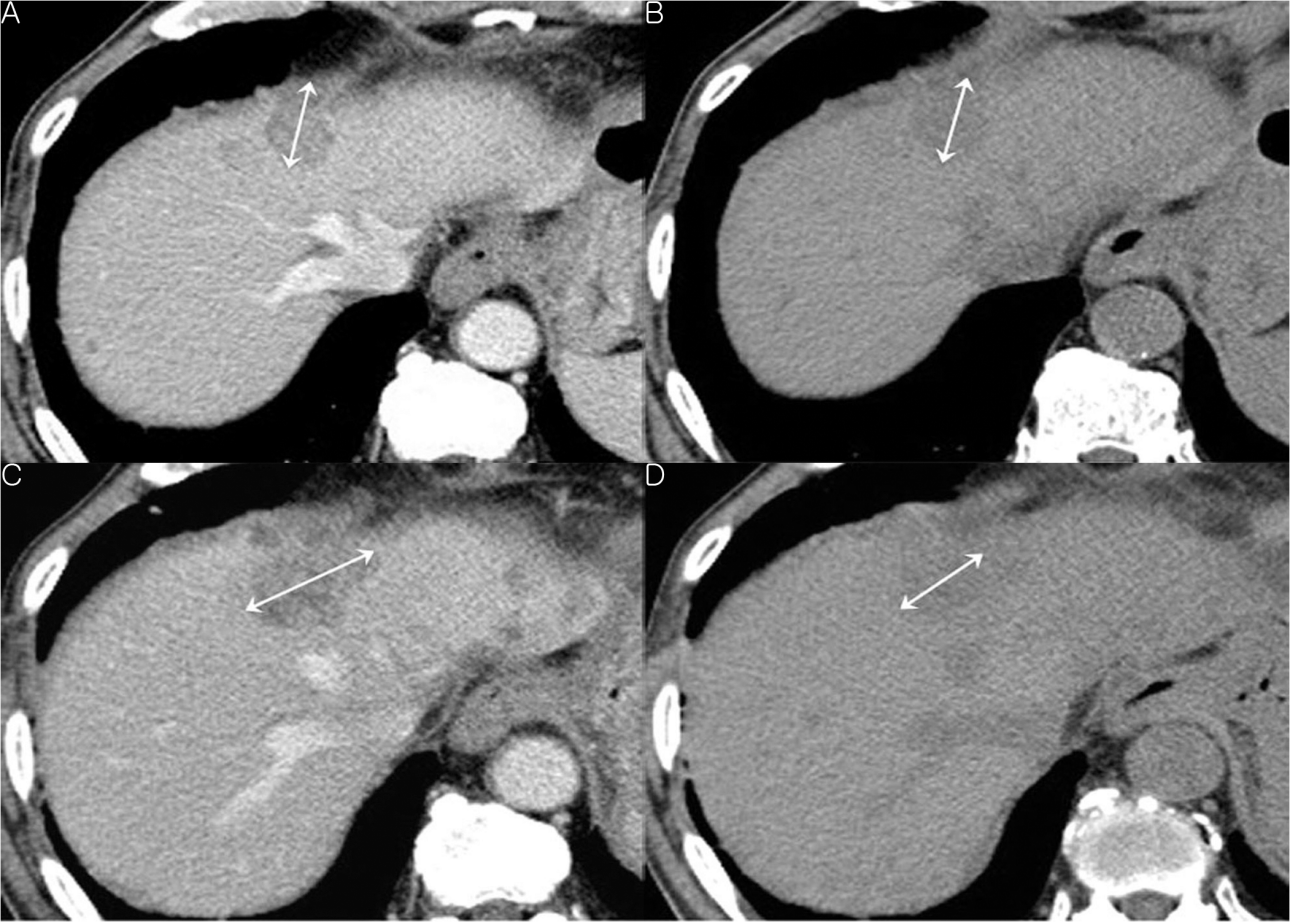
Seventy-two-year-old man who had known liver metastasis from gastric cancer. a. On the portal venous phase, hepatic metastasis is seen in segment 4 of the liver. b. The size of the lesion appears similar in the non-enhanced CT image. This lesion was measured as 30 mm both in the contrast-enhanced CT (a) and non-enhanced CT (b) images. (c, d) On follow-up CT after 3 months, the lesion had increased in size. It was measured as 47 mm (57% interval increase) on contrast-enhanced CT, which suggested progression of disease (c). However, on non-enhanced CT, the mass was measured as 32 mm (7% interval increase), which suggested stable disease.

In Group B, the accuracy of response evaluation was 72.2%, for reviewer 1 and 65.6% for reviewer 2 (Table 4) (Fig 3). There was no significant difference in diagnostic accuracy between the two reviewers, but there was a tendency toward overdiagnosis with reviewer 2 (Table 4). Interobserver agreement was moderate (κ = 0.591, 95% CI: 0.475–0.707).

**Table 4:** Accuracy of response evaluation based on the RECIST 1.1 criteria.

	Accurate diagnosis (95% CI)	Underdiagnosis (95% CI)	Overdiagnosis (95% CI)
**Reviewer 1**	72.2 (65.0–79.3)	18.5 (12.3–24.7)	9.3 (4.6–13.9)
**Reviewer 2**	65.6 (58.0–73.1)	15.9 (10.1–21.7)	18.5 (12.3–24.7)
**P value**	0.074	0.316	<0.001

Note: Numbers in parentheses are 95% confidence intervals.

## Discussion

Our results revealed that the diagnostic accuracy of NECT for the detection of newly developed hepatic metastasis in patients with colorectal and gastric cancer was 56.1–66.7% in per patient analysis and 52.6–56.8% in per lesion analysis. Per lesion diagnostic accuracy decreased significantly to 28.1–34.4% in the diagnosis of small metastatic lesions less than 1.5 cm in size. Furthermore, metastasis size measurements were significantly smaller on NECT compared with the reference standard CECT. In terms of diagnostic performance for evaluating treatment response based on RECIST 1.1 criteria, only 65.6–72.2% of patients were assessed correctly on NECT.

Because of the inherently low contrast resolution of the liver, optimal contrast enhancement is essential for the accurate diagnosis of focal hepatic lesions, and according to previous studies, at least 50 HU of hepatic enhancement is required to obtain high diagnostic quality hepatic CT images [13,14]. Although prior study reported that there was no significant difference in the assessment of focal hepatic lesions between NECT and CECT [15], a more recent study reported that PVP showed better diagnostic performance than LAP or NECT; 85% of liver metastases were detected on PVP whereas only 61% were detected on NECT, and there was no patient in whom all liver metastatic lesions were missed on PVP [16]. Our study showed similar results in that approximately half of the metastatic lesions and only about one-third of the metastatic lesions less than 1.5 cm in size were diagnosed on NECT. Based on these results, the diagnostic performance of NECT is inadequate for the detection of newly developed liver metastatic lesions, particularly small lesions. Hence, follow-up in patients who may develop new liver metastatic lesions with NECT might be inappropriate.

Nazarian et al [17] demonstrated that the sizes of hepatic metastatic lesions from colorectal cancer measured on precontrast images were significantly greater than those measured on postcontrast images for all lesion sizes. Because CM gradually diffuses into the extracellular space (ECS) of metastatic lesions after CM administration, the peripheral portion of the lesions might have presented with isoattenuation compared with the adjacent liver parenchyma, resulting in exclusion from size measurement [18,19]. In a previous study, spiral CT was used and whole liver scanning might have taken 25–30 seconds to complete [17]. Hence, postcontrast images might have been obtained closer to the equilibrium phase than the PVP, at least for part of the liver, resulting in more diffusion of CM into the ECS and smaller measured sizes of lesions on postcontrast images compared with precontrast images. In contrast, modern MDCT was used in our study, and the whole liver could be scanned in about 5 seconds. This enabled all images of the liver to be acquired within the PVP; thus, maximal CNR of hypovascular metastasis was obtained and lesion size could be measured before CM diffused into the ECS of hepatic metastasis. Both in lesions of all sizes and lesions less than 1.5 cm, the number of lesions was underestimated with NECT in our study. An explanation for this finding may be that low contrast resolution is inferior in NECT compared with CECT. Thus, in the study images, the central necrotic area—but not the peripheral viable portion—of the tumor could be differentiated from adjacent normal liver parenchyma, resulting in miscalculations of the lesion size on NECT.

In our study, 65.6–72.2% of patients were accurately assessed using RECIST 1.1 criteria during the follow-up of liver metastasis. This result was comparable with a previous study that revealed approximately 71% of patients showed concordant evaluation results between NECT and CECT [9]. These findings imply that overestimation or underestimation of treatment response may occur in one-third of patients, in whom alternative treatment options may not be considered or may unnecessarily be used. RECIST 1.1 criteria suggest that an optimal and consistent injection protocol of CM is important to accurately assess patients. If the administration of iodinated CM is contraindicated, an alternative imaging modality, such as NECT or MRI (with or without contrast enhancement), should be selected depending on the tumor type and anatomical location of each patient [10]. Based on our results, evaluation of metastasis in the liver with NECT might be inappropriate.

Ultrasound has been and is still widely used as a follow-up imaging modality because it is readily available and harmless to patients. However, sensitivity in the detection of liver metastasis was only about 40–55% with conventional US [3,20]. This result was probably due to operator dependency and possible blind spots due to lung, bowel, or patient obesity. Hence, US is not suitable as a sole follow-up imaging modality in patients with possible liver metastasis. Contrast-enhanced US is a possible alternative tool, and has shown similar diagnostic performance to CECT [20]. However, accessibility is limited because US CM does not have worldwide approval and requires dedicated software. Contrast-enhanced MRI has shown excellent diagnostic performance for liver metastasis with a sensitivity of about 76–88% [3,21]. However, patients with decreased renal function are considered to have a relative contraindication for contrast enhanced MRI because of the possibility of nephrogenic systemic fibrosis [22]. Although non-contrast MRI showed similar sensitivity to contrast-enhanced MRI in a prior meta-analysis [21], contrast enhancement is considered important both for detecting metastasis and, in particular, for characterizing focal liver lesions [6]. Furthermore, the current high cost, long examination time, and variable availability globally of MRI hinder its use widely as a first-line tool for the assessment of liver metastasis [6,10]. ^18^F-FDG PET can be another choice for the evaluation of liver metastasis without iodine contrast media. Recent meta-analysis demonstrates that ^18^F-FDG PET shows good accuracy for the detection of liver metastasis and can prevent unnecessary surgery [23,24]. However, the sensitivity of PET/CT markedly decreased after chemotherapy and many colorectal cancer patients with liver metastasis underwent chemotherapy before or after surgery, hence PET/CT could not completely replace contrast enhanced CT or MR [25].

This study has several limitations. First, this study was retrospective. Hence, the characteristics of the patients were heterogeneous, i.e., patients had different stages of gastric or colorectal cancer and underwent different treatments, and CT was performed at different intervals. However, we aimed to evaluate the diagnostic performance of NECT in the detection and evaluation of liver metastasis, and heterogeneity of patient characteristics might not have significantly influenced results but rather reflected actual clinical situations. Second, hepatic metastasis was not confirmed histologically. However, two experienced radiologists reviewed all imaging studies, clinical information, and laboratory results obtained over a follow-up period of at least 1 year to generate the reference standards, and we considered this sufficiently accurate for the diagnosis and evaluation of hepatic metastasis. Third, metastasis other than liver metastasis was not evaluated. Considering that the liver is the most common site of metastasis from gastrointestinal cancer and only about 23% of patients with liver metastasis have metastasis to other organs [26], evaluation of liver metastasis was considered critical in the overall assessment of disease status. However, future study is warranted involving the whole abdomen or body.

In conclusion, NECT showed inadequate diagnostic performances in both the detection of newly developed hepatic metastasis and the evaluation of the response of hepatic metastasis based on RECIST criteria in patients with gastric and colorectal cancer. Hence, contrast-enhanced CT with proper preparations or alternative imaging modalities such as MRI and US with/without contrast enhancement should be considered, even in patients with decreased renal function or hypersensitivity to iodinated CM.
